# Mapping mental life: the dimensional model as a framework for theory, practice, and research

**DOI:** 10.3389/fpsyg.2026.1737465

**Published:** 2026-02-25

**Authors:** Shlomo Mendlovic

**Affiliations:** The Shalvata Mental Health Center, Tel Aviv University, Hod HaSharon, Israel

**Keywords:** clinical supervision, dimensional model, meta-framework, psychological dimensions, psychotherapy process

## Abstract

Contemporary psychotherapy lacks an integrative framework that systematically connects behavior, cognition, emotion, experience, and being. The *Dimensional Model* of the Psyche addresses this gap by conceptualizing mental life as unfolding through five nested psychological dimensions- *action*, *thought*, *emotion*, *experience*, and *being*- within a descriptive, meta-theoretical framework governed by five operational principles that regulate the transitions between them. The present article introduces the full theoretical formulation of the model, articulating its conceptual foundations and dynamic principles. Clinical illustrations demonstrate how the model clarifies therapeutic processes and guides intervention. The *Dimensional Model* articulates a structured psychological continuum composed of qualitatively distinct yet dynamically co-present dimensions, offering an organizing language that bridges theory, clinical practice, and research.

## 1 Introduction

Theories in psychology, by their very nature, seek to organize the complexity of mental life into systems of principles capable of describing, conceptualizing, explaining, and guiding therapeutic practice (Fonagy and Target, 2003; Wampold and Imel, 2015). Yet time and again, it becomes evident that psychic reality, in its many forms, resists comprehensive formulation. Paradoxically, the very theories that aspire to portray mental life as a stable and coherent structure often reveal, through their own limitations, the insufficiency of theory to fully contain the psyche. Still, the limits of existing frameworks may become the source from which new concepts and paradigms in psychotherapy emerge, spurring conceptual innovation and moving the field forward (Frosh, 2016).

The partiality of theory within psychotherapy should not discourage the ongoing search for integrative frameworks capable of uniting the diverse bodies of knowledge that populate the field. Such frameworks can enrich dialogue among distinct traditions of thought (White, 2008) and may help bridge psychotherapy with adjacent domains of inquiry such as neuropsychoanalysis (Bazan and Detandt, 2017). Whether or not a “grand theory” is ultimately attainable, the importance of maintaining dialogue among perspectives, and of seeking coherence within multiplicity, remains indisputable (Orlinsky et al., 2017; Wallerstein, 1988).

The fragmentation of theory in psychotherapy is not merely an academic concern. As Wampold and Imel (2015) note, the “common factors” approach presupposes some underlying coherence of mind, one that has been empirically observed yet remains not fully defined at the theoretical level. Each framework nonetheless carries its own clinical derivatives, such as distinctive modes of case formulation in psychodynamic theory, and technical implications, for example in how interpretation is offered. Their importance is therefore considerable; yet the coexistence of multiple, partially overlapping models has hindered the development of a shared conceptual language capable of linking theory and practice. Despite consistent meta-analytic evidence of psychotherapy’s general efficacy, the field still struggles to identify the mechanisms that account for therapeutic change across approaches.

In response to this longstanding fragmentation, the *Dimensional Model* seeks to provide a coherent conceptual architecture capable of linking structure and process within a unified language of the psyche. Emerging from the need to integrate the diverse “languages of mind” (Henriques, 2004, 2017; Calhoun, 2004; Gintis, 2007; Melchert, 2016; Joseph, 2017; Reed, 2020; Swenson, 2023), the model conceives of mental life as a dynamically regulated system whose complexity can be understood through relational principles of order and transformation. It aims to integrate multiplicity and coherence not through reductionist unification but through an interdimensional logic that preserves differentiation within connectedness.

The *Dimensional Model* describes mental life as composed of five nested psychological dimensions- *action*, *thought*, *emotion*, *experience*, and *being*. The term “nested” does not imply a smooth continuum or hierarchical layering but rather a structured interdependence: each dimension provides the conditions for the emergence of the next while remaining qualitatively distinct. The nesting of dimensions reflects their structural interdependence, while their relational dynamics describe the ongoing interplay through which mental life unfolds in experience. Transitions between dimensions are not continuous gradations but patterned transformations, moments marked by shifts in the dominant mode of mental functioning. Each dimension expresses a particular orientation of the same underlying psychic process, differing in its mode of operation and temporal depth. Psychological phenomena thus arise not within any single dimension but through the dynamic interaction among them.

Seen from this perspective, the *Dimensional Model* situates major psychological paradigms within a shared structural horizon. Behaviorism’s focus on *action*, cognitive science’s emphasis on *thought*, interpersonal theory’s exploration of *emotion*, and phenomenology’s attention to experience and *being* may each be understood as illuminating distinct yet interrelated strata of psychological organization. The dimensional lens does not supersede these traditions; rather, it renders their points of convergence more conceptually explicit. In this sense, the model offers an organizing language through which structure and experience, mechanism and meaning, can be approached as reciprocally informing aspects of a dynamic system.

Importantly, the dimensional logic is not theoretically confined to any single school. Parallel constructs can be discerned across psychoanalytic, cognitive-behavioral, experiential, and process-based traditions, suggesting that the model may function less as a competing doctrine than as a transtheoretical grammar capable of supporting dialogue across theoretical boundaries.

The *Dimensional Model* is best understood as an epistemological framework grounded in an ontological hypothesis. Ontologically, it assumes that mental life exhibits a structured organization expressible through nested dimensions (*action*, *thought*, *emotion*, *experience*, and *being*). Epistemologically, it offers a disciplined language of observation through which psychological phenomena can be mapped, analyzed, and compared across theories and methods. It is therefore descriptive rather than metaphysical: it does not define what mind is, but delineates how mind appears and transforms within experience, communication, and therapeutic dialogue. At the same time, the model is not proposed as a closed “theory of everything.” It functions as a meta-theoretical scaffold, a framework for organizing existing findings without displacing their original assumptions. Its clinical and research applications are secondary derivatives: pragmatic illustrations of how dimensional principles can be observed, taught, and empirically examined. In this sense, the framework delineates the structure within which diverse psychological theories may coexist, rather than an all-encompassing system that supersedes them.

The aim of this paper is fourfold:

(1) to elaborate the five dimensions as distinct configurations of mental activity;
(2) to define their overall organization;
(3) to articulate the principles that govern transitions and interactions among them; and
(4) to explore the implications of this framework for clinical practice and research

## 2 The dimensions

A *dimension* refers to a distinct mode of mental functioning that organizes attention, processing, and engagement around a coherent psychological domain. The *Dimensional Model* proposes five such fundamental dimensions- *action*, *thought*, *emotion*, *experience*, and *being*- each representing a characteristic orientation of mental life.

The dimension of *action* concerns what a person does in the world. Not every behavior, however, belongs to the domain of the psyche. For the present purposes, *action* refers only to those behaviors that stand in reciprocal relation to the other dimensions (*thought*, *emotion*, *experience*, and *being*). Such actions are defined as intentional: they express an orientation shaped by inner states and directed toward explicit or implicit goals. Automatic or purely mechanical movements, lacking connection to the inner system of the mind, fall outside this scope.

Among the five dimensions, *action* is the most visible and externally accessible. We observe what others do, and others observe what we do. Yet because actions shape how we are perceived and how we perceive ourselves, the *action* dimension is not merely the behavioral output of the psyche but an active participant in its inner and interpersonal dynamics.

The dimension of *thought* refers to formulatable mental content, mental activity that can, at least in principle, be expressed in language. This definition does not deny the existence of unformulated (Stern, 1997) or pre-symbolic (Ogden, 1992) thinking, whether inaccessible to awareness (unconscious thought) or expressed in primitive, nonverbal form. Yet for the sake of conceptual clarity and clinical applicability, the *Dimensional Model* confines this domain to thought that can be meaningfully articulated.

In everyday life, *thought* organizes and guides much of our conduct. It serves as a bridge between action and emotion, transforming experience into reflection and reflection into guidance for behavior. Because formulated thought can be shared, it allows internal processes to become objects of dialogue and examination, linking individual understanding to the shared language of the interpersonal world. Still, communication itself does not originate in thought but in emotion; thought gives emotional life its form and intelligibility, rather than its expressive power.

The dimension of *emotion* encompasses our formulated feelings directed toward people, objects, situations, or ideas that lie beyond the immediate boundaries of the self. It refers to what we consciously feel about another person, thing, or circumstance.

While countless affective states arise within us without clear formulation, the emotional dimension concerns those feelings that can be recognized, named, and expressed.

Focusing on articulated emotion highlights how affect participates in meaning-making and relational life.

*Emotion* constitutes a central fabric of our existence. Our feelings toward friends, family, community, places, objects, and even abstract ideals shape both our inner world and our social reality. In nearly every encounter, asking “What do I feel toward the other or toward this?” yields an identifiable emotion or mix of emotions. At times, such as in love, anger, or grief, emotion carries decisive psychological weight, temporarily dominating the other dimensions of the psyche and organizing experience around its own logic of attachment and significance. As the primary bridge between inner life and the external world, emotion communicates what is lived inwardly through embodied and relational expression, even before words are found.

The dimension of *experience* turns inward. It refers to what we live through within ourselves—an inner current of felt life that is not necessarily formulated in words or concepts. *Experience* is the dimension of immediacy: the felt presence of being oneself at a given moment. Because it unfolds within the intrapsychic field, it often resists articulation; language trails behind it, leaving much of its content indeterminate or only partly symbolized.

*Experience* is continuously present in our lives. We may cease to act, to think in words, or to feel toward others, but we cannot cease to experience ourselves. This unbroken flow of inward life serves as a kind of compass, orienting us toward meaning even when we cannot yet express or follow it. Though private and elusive, the *experiential* dimension anchors our sense of vitality and coherence. It provides the felt ground of authenticity from which the other dimensions emerge and return, yet each moment of experience is transient, arising and dissolving within the ongoing movement of inner life.

The dimension of *being* represents the deepest and most elusive stratum of the psyche. While aspects of identity, meaning, and value may express this dimension, being is not reducible to them. The term is intentionally retained to designate the ontological ground from which such structures emerge, rather than the structures themselves. It may be defined as the latent and enduring dimension that shapes the range of psychic possibility, the underlying structure that determines what forms of *experience*, *emotion*, *thought*, and *action* can emerge. *Being* is to the psyche what the musical key is to melody, or the canvas to painting: it does not dictate the specific content of mental life but defines its field of potential expression.

Although rarely an object of direct awareness, being is disclosed through the tacit sense of continuity that allows a person to experience themselves as the same across time and circumstance. It typically recedes from attention precisely because it functions as the condition of psychological coherence rather than as one of its contents. Only when this continuity is threatened- through crisis, transformation, or existential questioning- does being move from background to figure. Thus, the question “Who are you, really?” does not seek descriptive attributes but gestures toward the ontological ground that silently organizes them.

Table 1 summarizes the definition of the dimensions, their orientation, guiding focus, phenomenological qualities, and an illustrative utterance exemplifying each dimension.

**TABLE 1:** The dimensions.

Dimension	Definition	Orientation	Guiding focus	Phenomeno-logical qualities	Illustrative utterance
Action	Intentional behavior directed toward a goal; what a person does in the world.	Outward, world-directed	What am I doing?	Observable, purposive, externally mediated	“I’ve called her today and tell her what I think.”
Thought	Formulatable mental activity; language-based reflection and reasoning	Bridging between action and emotion	What am I thinking?	Symbolic, reflective, communicable	“I keep wondering why I reacted that way yesterday”
Emotion	Formulated feeling directed toward people, objects, or situations beyond the self	Relational, object-directed	What do I feel toward the other (or toward this)?	Affective, expressive, meaning-laden	“I’m angry at him”
Experience	Felt inner life not necessarily articulated in words; the immediate sense of living through oneself.	Inward, self-directed	What am I experiencing right now?	Momentary, pre-verbal, inwardly felt	“There’s this tightness in my chest that I can’t quite describe”
Being	Latent, enduring foundation that defines the range of psychic possibility and identity.	Foundational, existential	Who am I?	Silent, stable, all-encompassing	“I just feel like I’m not the person I used to be”

Taken together, the five dimensions outline a coherent architecture of mental life. Although conceptually distinct, they are continuously interwoven in lived experience, dynamically shaping one another. At any given moment, one dimension may become more prominent, organizing the psyche according to its particular logic. In their overall arrangement, the dimensions form what may be termed a *Cascade of Ddimensions*- an ordered depth structure through which mental life is both generated and transformed while maintaining coherence.

## 3 The cascade of dimensions

The five dimensions may be viewed as forming a nested architecture of the psyche. Each provides the conditions for the emergence of the next while remaining qualitatively distinct in its mode of functioning. This nesting expresses the structural dependency of mental life: higher dimensions presuppose lower ones (e.g., thought presupposes intentional action).

Yet mental life is not a static hierarchy. The dimensions sustain a dense web of reciprocal relations in which each can influence, and be influenced by, any other. *Thought* may give rise to *action* (“I decided to call her because I thought it was important to be honest”), to *emotion* (“I think he’s struggling, and that makes me feel compassion”), to *experience* (“I realize I acted wrongly, and now I feel guilty”), or even to *being* (“I always think like a therapist; that’s simply who I am”). Likewise, each dimension may emerge as the outcome of processes originating in the others. The psyche thus functions as a dynamic network rather than a fixed chain of command, with each dimension serving as both potential cause and consequence.

Still, as complexity increases, theoretical clarity requires an orienting principle, one that can describe the typical direction of mental flow without denying its multidirectionality.

The *dimensional cascade* denotes this principle of organization: a conceptual ordering that outlines the most common pattern through which psychic activity unfolds.

Importantly, the cascade is not intended as a dogmatic or exclusive ordering of mental life. Alternative sequences are theoretically conceivable and may reflect different epistemological entry points into the psyche. For example, phenomenological traditions often begin with lived experience, whereas behavioral perspectives may privilege action. The present formulation is therefore offered as a heuristic map rather than an ontological claim, designed to clarify the most typical direction of psychological transformation while preserving the multidirectional character of mental processes.

The cascade begins with *being*, the existential foundation that shapes the range of psychic possibility; proceeds through *experience*, the inward sense of unformulated feeling; moves outward to *emotion*, the formulated affect directed toward other or an object; continues to *thought*, the conceptual articulation of meaning; and culminates in *action*, the intentional realization of mind in the world.

Each dimension thus derives from the one that precedes it. *Being* gives rise to *experience*, much as Winnicott’s *going-on-being* provides the basis from which a sense of self can emerge (Winnicott, 1960). *Experience* gives rise to *emotion*, echoing Kohut’s (1971) view that affect is secondary to the experiential state that precedes it. *Emotion* gives rise to *thought*, in line with Bion’s claim that when the link to a real object is absent or damaged, thought cannot develop (Bion, 1962). Finally, *thought* gives rise to *action*, for intentional behavior presupposes conceptual representation.

The proposed ordering of the cascade (placing, for instance, experience before emotion or emotion before thought) finds support in converging evidence from developmental observation, neuropsychological organization, and psychoanalytic theory. A full exposition of these foundations exceeds the scope of this paper. Yet beyond theoretical justification, the most compelling reason for adopting this formulation lies in its intuitive plausibility. As Whitehead (1920) (1920/1919) observed, “The guiding motto in the life of every natural philosopher should be, ‘Seek simplicity and distrust it”’ (p. 163). The dimensional cascade seeks precisely such tempered simplicity: a map of the psyche that remains intelligible while honoring the complexity it seeks to describe.

While the cascade is presented as a heuristic ordering, its purpose is explanatory rather than merely descriptive: Its value lies not in asserting a single necessary sequence, but in providing conceptual coherence that allows diverse theoretical perspectives to be situated within a shared structural framework.

## 4 Principles of dimensional dynamics

Drawing on the five psychological dimensions (*action*, *thought*, *emotion*, *experience*, and *being*) arranged in a tendential sequence (the *dimensional cascade*), we can now outline the principles that govern transitions among them. These principles describe how shifts between dimensions unfold both in everyday mental life and within the therapeutic encounter.

Together, the five principles depict the dynamic organization of the psyche: how transitions occur within the individual mind and between minds in intersubjective exchange. They account for the movement of mental life across dimensions, the coordination of psychological states between individuals, and the balance whose maintenance underlies mental health. The dimensional arrangement should therefore not be understood as a fixed hierarchy, but as a dynamic structural organization in which each dimension both emerges from and recursively reshapes the others over time.

### 4.1 Dominance

The *Principle of Dominance* holds that, in any given moment, attention is organized around a single dominant dimension of functioning. This principle mirrors the organization of the nervous system, where conscious awareness can sustain only one coherent representation at a time, never multiple or contradictory ones (Mashour et al., 2020).

Of course, the psyche is always distributed across all dimensions. Even now, as you read these lines, your mind is simultaneously active in several domains: in *action* (the physical act of reading), in *thought* (grasping the author’s intent), in *emotion* (feeling something toward the text or its author), in *experience* (sensing an inner state- perhaps curiosity, irritation, or absorption), and in *being* (inhabiting your enduring professional identity as a clinician or scholar). Yet focused awareness can occupy only one dimension at a time.

Much like Rubin’s famous vase illusion, where one perceives either two faces or a vase, but never both simultaneously, consciousness alternates between possible configurations.

We cannot hold two incompatible Gestalts at once, just as we cannot be fully present in more than one psychological dimension in the same moment.

The *Principle of Dominance* is evident both in everyday mental life and in psychotherapy. Activating a cognitive operation, such as performing repeated subtraction (e.g., 100-7-7…), can redirect attention away from an affective state of anger. This mechanism underlies many anger-management interventions, which train participants to shift from *emotion* to other dimensions, typically *thought* (e.g., spelling a long word backward) or *action* (e.g., performing a repetitive physical task such as tossing a yo-yo) (Denson et al., 2012; Kjærvik and Bushman, 2024). Such redirection rests on the *Principle of Dominance*: by mobilizing another mode of functioning, the psyche reorganizes itself, thereby reducing the dominance of the emotional state.

### 4.2 Continuity

The *Principle of Continuity* states that transitions within the psyche occur between *adjacent dimensions*, unfolding sequentially rather than through abrupt leaps. Movement along the dimensional cascade, from *being* to *experience*, from *experience* to *emotion*, from *emotion* to *thought*, from *thought* to *action*, or in reverse, proceeds in sequence, without skipping a level. In other words, the psyche evolves through successive transitions in which each mode of functioning arises from the one before it and conditions the emergence of the next. Mental life progresses through transitions that mediate and integrate what precedes with what follows.

For example, when moving from *emotion* (“What do I feel toward the other?”) to *action* (“What do I do with the other?”), the transition necessarily passes through *thought* (“What meaning or decision arises from my feeling, and what action does it entail?”).

Likewise, when moving in the opposite direction, from *action* (“What did I do with the other?”) to *emotion* (“What do I feel about it?”), the shift unfolds through *thought* (“What do I think about what I did, and what feeling follows from that thought?”).

This principle rests on a developmental and phenomenological premise shared across major psychological traditions: transformations within the psyche unfold through neighboring modes of functioning rather than through abrupt leaps. In psychodynamic theory, the Principle of Continuity can be traced from Freud’s (1894) notion of affect transformation to Bion’s (1962) description of converting beta elements into alpha elements, both depicting a sequential, metabolizing process rather than a discontinuous shift (Riolo, 2007). Developmental and cognitive models, from Piaget’s stages (Quartz, 1999) to Edelman’s Neural Darwinism (Seth and Baars, 2005) likewise conceive psychological organization as a graded coordination of perceptual, emotional, and conceptual systems. In phenomenology, Merleau-Ponty’s (2012) “continuous intentional arc” (1945) and Heidegger’s account of Dasein as temporal unfolding both affirm that being, feeling, and acting are contiguous expressions of a single coherent movement of mind (Heidegger, 1962).

Everyday decision-making illustrates the *Principle of Continuity* with particular clarity.

When an individual acts deliberately, the psyche rarely leaps between states; rather, it traverses a sequence of adjacent transitions (*experiencing*, *feeling*, *thinking*, and *acting*), each shaping the next. Contemporary research on decision-making supports this conception. Neurocognitive models such as Damasio, 1996 Somatic Marker Hypothesis suggest that adaptive choice depends on a graded integration of bodily, emotional, and cognitive signals rather than on discrete computation. Dual-process theories (Kahneman, 2011; Evans, 2008) likewise describe reasoning as a continuous interaction between intuitive and reflective systems. Taken together, these findings suggest that coherence arises from the psyche’s capacity to transform *experience* into *emotion*, *emotion* into *thought*, and *thought* into intentional *action*.

This sequential description should be understood as the typical architecture of psychological transformation rather than as an invariant rule. Clinical change may at times appear to involve non-adjacent shifts; however, such moments often reflect rapid integrations in which intermediate processes remain phenomenologically implicit rather than structurally absent. The principle of continuity therefore describes the psyche’s underlying organization even when experiential change appears sudden.

In clinical work, the *Principle of Continuity* is vividly expressed through the operation of defense mechanisms, which disrupt the psyche’s integrative movement linking affect, thought, and symbolization. As Fonagy and Target (1996) observe, defensive exclusion entails “a breakdown in the continuity of mental experience,” interrupting this integrative process and fragmenting the links that connect emotion, cognition, and meaning. In acting out, the individual moves directly from *experience* to *action*, bypassing *emotion* and *thought*, so that the feeling is enacted rather than symbolized. In intellectualization, movement halts within *thought*, severed from its emotional and experiential ground. In repression, the passage is blocked even earlier, preventing *experience* from transforming into *emotion*. Each defense thus represents a failure of transition between adjacent dimensions. Therapeutically, restoring these links (e.g., enabling *emotion* to emerge from *experience*, or *thought* from *emotion*) re-establishes the psyche’s natural continuity and allows meaning, rather than impulse, to govern action.

### 4.3 Equilibrium

The *Principle of Equilibrium* holds that the psyche continuously regulates the balance among its dimensions, adapting to both internal and external demands. Psychological equilibrium is never static; it is inherently contextual and shifting. At any given moment, the psyche maintains a dynamic balance among its five dimensions, *action*, *thought*, *emotion*, *experience*, and *being*, which reorganizes in response to situational pressures or inner tensions. In a therapeutic encounter, for instance, a discussion about medication decisions may evoke a configuration dominated by thought, where analytical reasoning prevails; yet when the dialogue turns to the emotional meaning of a diagnosis, the equilibrium often shifts toward deeper emotional and experiential layers, where feelings are not only expressed but inwardly lived, and the therapist’s attunement helps sustain the emerging experience.

Stimulation that provokes inter-dimensional transitions may originate externally or internally, from another person, an evocative event, or an emergent impulse within the psyche itself. Gentle or transient stimulations induce soft, reversible transitions that momentarily reshape dimensional balance before returning to a habitual state. Intense or prolonged stimulations, such as traumatic or life-altering experiences, may produce deeper transitions that disrupt and reorganize the psyche’s equilibrium, often leading to a new configuration that may be either adaptive or maladaptive, yet bears the enduring imprint of transformation.

The *Principle of Equilibrium* is evident both in everyday life and in psychotherapy. In daily experience, it manifests as the subtle yet constant regulation of balance among the dimensions. This dynamic is especially visible in infancy. As Stern (1985) described, the minute exchange of signals between infant and caregiver generates micro-movements within the psyche, tiny oscillations between experience and emotion that sustain vitality, regulation, and connection. Crucially, Stern’s observations show that changes in psychological equilibrium must occur on a moment-to-moment basis and with exquisite delicacy: when transitions are too abrupt or intense, regulation collapses; when they remain finely tuned and responsive, integration deepens. The psyche, like the dyadic field between infant and mother, reorganizes not through force but through sensitivity (i.e.,- through the smallest possible perturbations that preserve coherence while enabling transformation).

In psychotherapy, the same principle operates through interpersonal resonance.

The therapist’s intervention, whether interpretive, empathic, or experiential, acts as a stimulus that gently perturbs the system of dimensional relations, momentarily shifting the patient’s habitual equilibrium. A reflective interpretation may reorient balance toward *thought*, while an empathic attunement invites *emotion* or *experience* to re-emerge. When the intensity of the stimulus exceeds the patient’s capacity for regulation, however, equilibrium may collapse, resulting in a therapeutic rupture rather than integration (e.g., Eubanks et al., 2018). When therapeutic contact provides sufficient stability and containment, these micro-perturbations instead accumulate over time into deeper reorganization, allowing the psyche to establish a renewed equilibrium in which previously dissociated dimensions can coexist in greater harmony (see, for example, Kernberg et al., 2012).

### 4.4 Mutual attraction

The fourth principle governing the relations among dimensions is the *Principle of Mutual Attraction*: when two individuals meet, each tends, often unconsciously, to draw the other toward the equilibrium of their own dimensional state. Put simply, when two people occupy different dimensional states and encounter one another, each tends to draw the other toward their own dominant dimension.

The *Principle of Mutual Attraction* operates across all dimensions. When someone attacks us (*action*), we instinctively defend ourselves physically (*action*); when a friend poses a riddle (*thought*), we are drawn into reasoning (*thought*); When a partner says “I love you” (*emotion*), it evokes a feeling in us (*emotion*); when another person turns inward and dwells in their inner world (*experience*), our attention often follows inward (*experience*); and when we encounter someone whose being radiates calm and groundedness, our own sense of being tends to harmonize with theirs.

In everyday life, the *Principle of Mutual Attraction* is principle quietly operates whenever emotional or mental states synchronize between people. A calm interlocutor can gradually slow the speech, breathing, and thought of an anxious partner; an agitated atmosphere in a group can quickly mobilize action or defensiveness among its members. These are not instances of content being “passed” from one person to another, but of equilibrium being co-regulated, as each person’s dimensional configuration subtly re-tunes itself in relation to the other’s.

In psychotherapy, the *Principle of Mutual Attraction* can be understood as a dynamic of reciprocal regulation that underlies all relational processes. Conceptually, it resonates with the psychoanalytic notion of projective identification as a bidirectional communicative process (Ogden, 1982), and with later intersubjective and relational theories that view the therapeutic dyad as a system of mutual influence (Stolorow et al., 1994; Mitchell, 2000). Empirically, similar dynamics have been described in research on affective attunement and mutual regulation (Stern, 1985; Tronick, 1989), as well as in neuropsychological studies of mirror systems and empathic resonance (Gallese, 2003). Across these perspectives, therapeutic interaction is understood not as a unidirectional intervention but as a co-regulated field, in which each participant’s dimensional configuration continuously adjusts in response to the other’s. Within the framework of the dimensional model, this corresponds to a constant modulation of balance among *action*, *thought*, *emotion*, *experience*, and *being*, through which shared meaning and transformation emerge.

### 4.5 Flexibility

The fifth principle, the *Principle of Flexibility*, holds that the capacity to move freely between dimensions constitutes both a sign and a mechanism of psychological adaptability. Throughout the psychoanalytic tradition, mental health has been associated with the ability to shift fluidly among different modes of psychic organization. For Freud (1900), this was reflected in the free mobility of psychic energy; for Bion (1962), in the mind’s capacity to oscillate between modes of thought; for Winnicott (1971), in the spontaneity that allows play between inner and outer reality; and for Bromberg (1998), in the flexible movement among self-states that transforms dissociation into integration. Within the *Dimensional Model*, the *Principle of Flexibility* extends this long-standing idea: the greater the fluidity of movement across the five dimensions, the greater the individual’s adaptability, coherence, and psychological well-being.

Table 2 presents the five principles of dimensional dynamics.

**TABLE 2:** The five principles of dimensional dynamics.

Principle	Definition
Dominanace	At any given moment, attention is organized around a single dominant dimension
Continuity	Transitions occur gradually between adjacent dimensions
Equilibrium	The psyche maintains a dynamic balance among its five dimensions, which can be temporarily reorganized in response to internal or external influences
Mutual attraction	when two individuals meet, each tends, often unconsciously, to draw the other toward the equilibrium of their own dimensional state
Flexibility	The capacity to shift between dimensions both reflects and promotes psychological adaptability and health

## 5 Implications

Having outlined the five principles that govern transitions among the psychological dimensions, the discussion now turns to their broader implications.

The *Dimensional Model* is intended to provide a shared descriptive language that clarifies how different perspectives intersect within the same psychological field. Its value therefore lies not in prescribing new techniques, but in illuminating the underlying structure that underlies clinical process and informs research.

The discussion that follows outlines two principal avenues of application. First, at the clinical level, the model offers a framework for observing, conceptualizing, and guiding therapeutic interaction through the dynamic movement among dimensions. Second, at the research level, it suggests new ways to operationalize and measure the transitions that constitute mental life, bridging experiential and behavioral data within a single dimensional continuum.

### 5.1 Implication in clinical practice

The dimensional model provides a clinically accessible method for analyzing and refining psychotherapeutic work. By translating complex psychodynamic principles into a concise and structured model, it allows therapists to observe the unfolding of therapeutic dialogue in terms of transitions between five psychological dimensions. Each utterance in the clinical exchange can be viewed as representing one dominant dimension, and the progression of these utterances reflects the movement of the therapeutic process itself.

This approach enables the therapist to identify how the dialogue evolves, whether it progresses smoothly through adjacent dimensions or exhibits abrupt transitions that may indicate defensive maneuvers, ruptures, or missed opportunities for attunement. The framework is guided by the five governing principles [*dominance* (which states that only one dimension is active at a time); *continuity* (which posits that movement occurs between adjacent dimensions); *equilibrium* (which describes the dynamic balance among them); *mutual attraction* (which captures the intersubjective pull between therapist and patient); and *flexibility* (which reflects psychological adaptability and health)].

By mapping clinical material through these principles, therapists can gain a clearer view of the structure and rhythm of their interventions. Breaks in continuity or dominance, for example, can signal moments where the therapist’s focus diverged from the patient’s experiential position. Conversely, alignment across dimensions can reflect depth, resonance, and therapeutic growth. The model thus provides a precise language for describing therapeutic processes, enabling the therapist to reflect not only on what was said, but on how each intervention positioned itself within the unfolding psychological process.

In clinical supervision and training, the dimensional perspective offers more than a practical framework. It establishes a shared conceptual language for articulating the complexity of therapeutic encounters. By mapping the shifting configurations of *action*, *thought*, *emotion*, *experience*, and *being* within the session, it grounds reflective dialogue in a coherent systemic logic. This coherence bridges theory and technique, enabling supervision to move beyond procedural correction toward an exploration of the evolving psychological field itself. In this way, the dimensional model transforms psychodynamic understanding into a structured yet flexible method of reflective inquiry, one that is teachable, replicable, and anchored in lived clinical phenomena. It thus provides a fertile basis for implementing deliberate practice (Hilsenroth and Diener, 2017; Rousmaniere et al., 2017) in psychotherapeutic practice and supervision (Mendlovic, 2025) supervision, responding to recent calls for frameworks that operationalize reflection and growth within the everyday ecology of supervision (Berning et al., 2024; Clements-Hickman and Reese, 2020).

The following four clinical vignettes illustrate how the operational principles of the Dimensional Model can guide moment-to-moment understanding in psychotherapy. All clinical vignettes are fictional and constructed solely for illustrative purposes. They are not drawn from, nor do they quote, any real clinical case or therapeutic session. Accordingly, no patient data or identifying details are included. Each vignette presents a brief segment of patient discourse, followed by two therapist responses (Therapist 1 and Therapist 2). The first response exemplifies alignment with the principle under discussion, while the second deliberately departs from it. Together, these contrasting interventions demonstrate how adherence to- or deviation from- the relevant dimensional principle shapes the session’s depth, continuity, and vitality.

For the sake of illustrative simplicity, all examples focus on the dimension of *experience*, which provides a common ground for demonstrating the model’s clinical logic.

#### 5.1.1 Example 1 – principle of dominance

##### 5.1.1.1 Patient

It’s hard for me… I feel bad… with myself… like… I don’t know… something inside doesn’t sit right… it’s not a constant feeling for me, but in the past few days it’s been stronger… everything intensified after I was told that I wouldn’t be promoted at work… I was disappointed… I started asking myself why I’m trying so hard… whether I’m really at peace with my work… and so, in the past few days, this feeling has been accompanying me, especially when I think about work… a kind of inner sense of meaninglessness… It feels like there’s a concentrated inner sense of “what’s the point,” a felt meaninglessness that you’re encountering within yourself…

##### 5.1.1.2 Therapist 1

It feels like you’re describing a very sharp, clear experience… concentrated… of “what’s the point?” of “where is this going?”… an experience of meaninglessness—one you’re having with yourself, and it’s not a pleasant one.

##### 5.1.1.3 Therapist 2

You’re saying a lot of things… that you’re feeling a kind of loss of direction, that you’re disappointed with your workplace for not promoting you… and then there’s a dilemma—about how to go on from here, and even questions like what should I do?

##### 5.1.1.4 Analysis

The patient’s discourse is centered in the dimension of *experience*, expressing an inward, felt sense of meaninglessness. Therapist 1 stays within this dominant dimension, echoing the patient’s experiential field and enabling deeper exploration toward *being*. Therapist 2 violates the *Principle of Dominance* by shifting prematurely to *thought* and *action*. This cognitive reframing organizes the material but breaks alignment with the patient’s lived state, reducing experiential depth and emotional contact. Therapeutic attunement therefore depends on sustaining attention within the patient’s dominant dimension before facilitating transitions.

#### 5.1.2 Example 2 – principle of continuity

##### 5.1.2.1 Patient

It happened to me again… during the team meeting… the conversation among my colleagues was so lively and meaningful, and I had important things to say… but… I was paralyzed… inside, I felt I just couldn’t speak… as if it had nothing to do with what was happening in the room… it was me, with myself, feeling that I simply couldn’t talk… I went silent… and was left with this feeling that I had something to say, but what stopped me was myself… maybe like a car that can’t start moving because its handbrake is locked… that’s how it felt.

##### 5.1.2.2 Therapist 1

You know, even though you say it was “you with yourself,” I imagine there was a whole interaction happening there between you and your colleagues… something in the room that closed you off… can you say something about what was happening there? About what you felt toward the others?

##### 5.1.2.3 Therapist 2

I imagine a lot was going through your mind at that moment… many thoughts you were preoccupied with… maybe you’ve developed a kind of cost–benefit mechanism, calculating when it’s worth speaking up and when it isn’t?

##### 5.1.2.4 Analysis

The patient speaks from the dimension of *experience*, describing an immediate, embodied paralysis- a felt suspension of agency. Therapist 1 gently opens the relational field, maintaining *Continuity* by moving through an adjacent dimension (from *experience* toward *emotion*) without disrupting the patient’s state. Therapist 2, however, breaks the *Principle of Continuity* by moving directly to thought, offering a cognitive explanation that interrupts the emotional flow. The shift fragments the immediacy of the moment and distances the patient from the embodied struggle that seeks articulation within the relational field.

#### 5.1.3 Example 3 – principle of equilibrium

##### 5.1.3.1 Patient

I feel terribly alone… now… here… in the room… I come because I want to change something in my life, not to feel so alone… and even here I feel alone… even here I feel alone.

##### 5.1.3.2 Therapist 1

A powerful sense of loneliness… a painful experience that seems to settle in… and perhaps, to understand it and what it does to you, I want to ask whether, together with this feeling, something is also stirred toward me- something you feel right now, here, in the room, toward me?

##### 5.1.3.3 Therapist 2

A painful experience of loneliness… that you’re feeling even here… an experience that, the more you feel it, the more it seems to settle in and deepen.

##### 5.1.3.4 Analysis

The patient’s speech in anchored in the dimension of *experience*, voicing an immediate sense of isolation. Therapist 1 offers a subtle, constructive stimulus- a question that invites relational reflection and opens a path from *experience* to *emotion*. This intervention restores *equilibrium*, allowing movement while keeping contact. Therapist 2 mirrors the loneliness accurately but fails to introduce any activating element that could alter the state. The absence of stimulus maintains contact but prevents transformation, leaving the patient confined within the same painful experience and thereby violating the *Principle of Continuity.*

#### 5.1.4 Example 4 – principle of mutual attraction

##### 5.1.4.1 Patient

It’s like a black hole… I feel it here… in the center of me… in my stomach… every time I recall that moment—the horror of it—I don’t think or feel; I’m just pulled into this black hole… and I have nothing wise to say about it… I, who can always describe and explain, suddenly fall into this nothingness… and it also feels terribly lonely.

##### 5.1.4.2 Therapist 1

It’s happening here too, now, in the room… this “black hole” is here with us… it’s hard to describe, to think, to feel, to explain… it’s as if we are together inside this “black hole”- and maybe, in some way, that keeps you from being alone there.

##### 5.1.4.3 Therapist 2

I’m thinking about the process… about how this happens to you, and I think it’s a meaningful process- one that, in the end, leaves you alone.

##### 5.1.4.4 Analysis

The patient speaks from the dimension of *experience*, at the edge of articulation, where thought, emotion, and language begin to collapse. Therapist 1 embodies Principle 4 (*Mutual Attraction*) by entering the patient’s experiential field and sharing it empathically, co-creating a moment of connection that begins to counteract isolation.

Therapist 2, however, analyzes the process from an external vantage point, maintaining cognitive distance and failing to join the patient’s affective world. This breaks *Mutual Attraction*, disrupting dyadic regulation and leaving the patient alone within the very experience that longs for contact.

Across these clinical examples, the *Dimensional Model* reveals itself as both descriptive and operative: it captures how therapists and patients co-regulate transitions among dimensions in real time. The same logic that organizes therapeutic dialogue can thus be examined empirically, through patterns of movement, synchrony, and alignment within and between minds.

### 5.2 Implication in research

Systematic coding of therapeutic dialogue has become a cornerstone of psychotherapy research, enabling fine-grained examination of therapeutic processes and mechanisms of change (e.g., Pereira et al., 2025). The MATRIX (Mendlovic et al., 2017) exemplifies such a framework: a theory-based and empirically validated tool designed to map the phenomena occurring within the therapeutic encounter. It is structured along two intersecting axes, the focus of observation (patient, therapist, dyad) and the dimension of inquiry (potential, content, relation), yielding nine possible configurations that together capture the dynamics of clinical dialogue.

Across several studies, the MATRIX has demonstrated strong reliability and predictive validity. Synchrony between patient and therapist codes was shown to distinguish successful treatments, with increasing convergence over time associated with positive outcomes (Mendlovic et al., 2019). Subsequent analyses revealed that congruence within the potential dimension accounted for most of the variance in treatment results (Bar et al., 2021), underscoring its theoretical and clinical significance. More recent work extended the MATRIX to quantify discursive richness and to identify ineffective “out-of-matrix” interventions, both yielding predictions of alliance and symptom change (Bar et al., 2023, 2025). Together, these findings illustrate how dimensional coding can advance the empirical study of psychotherapy by operationalizing the transitions and balances that define its inner logic.

The MATRIX is one example among a range of systematic process-research tools in which psychotherapy sessions are transcribed, coded, and analyzed according to defined theoretical models. These frameworks have sought to operationalize the moment-to-moment unfolding of treatment. Such are the Core Conflictual Relationship Theme (CCRT), which maps recurring relational patterns of wish–response of other–response of self; the Structural Analysis of Social Behavior (SASB), which codes interpersonal exchanges along axes of love, control, and autonomy; the Assimilation of Problematic Experience Scale (APES) and the Experiencing Scale, which quantify levels of emotional processing; and systems such as the Psychotherapy Process Q-Sort, Narrative Process Coding, and Rupture-and-Repair analyses, each highlighting different facets of therapeutic interaction (for extensive review, see Luo and Levendosky, 2025).

Like these instruments, the *Dimensional Model* seeks to describe the underlying organizational structure of mental processes. However, it extends beyond the analysis of therapeutic discourse, offering a parsimonious and broadly applicable framework for conceptualizing psychological functioning. Its advantages lie in its clarity, integrative potential, and ease of use: the model captures the complexity of mental life through five nested and hierarchically related dimensions (*action*, *thought*, *emotion*, *experience*, and *being*) which can be applied consistently across clinical, research, and theoretical contexts.

The following propositions illustrate how the model’s principles can be translated into falsifiable research hypotheses. To translate the *Dimensional Model* from conceptual framework to testable system, future studies should operationalize its principles as measurable process variables. Several hypotheses are directly derivable from the model. For instance, violations of the *Principle of Continuity* (abrupt transitions skipping adjacent dimensions) may predict therapeutic ruptures or defensive enactments, while greater dimensional flexibility, quantified as the range and fluidity of transitions within sessions, should correlate with better therapeutic outcomes. Similarly, alignment between patient and therapist dominant dimensions could serve as an index of attunement and alliance strength, whereas persistent mismatches might forecast dropout or stagnation. These propositions invite the integration of process coding, linguistic analysis, and computational modeling to trace dimensional dynamics over time. Embedding such metrics within ongoing clinical trials could provide the empirical grounding necessary to evaluate, and refine, the *Dimensional Model* as both explanatory and predictive framework.

## 6 Discussion

The five dimensions of the psyche together constitute the *Dimensional Cascade*, a dynamic system governed by five operational principles. This integrative framework offers both clear advantages and notable limitations that warrant consideration.

### 6.1 Advantages

The *Dimensional Model* introduces a genuinely systematic language for describing mental activity without collapsing the complexity of the therapeutic encounter. Its main advantage lies in structural precision: it makes visible the transitions that usually remain implicit in psychodynamic formulations. This precision allows for empirical coding and comparison across cases, something few meta-theoretical models achieve.

Yet the model’s greatest contribution is methodological rather than doctrinal: it provides a grammar of psychological movement that different theoretical schools can use without abandoning their own metaphors. In this sense, its integrative power rests not on theoretical synthesis but on shared observability, a common map for studying how mind and meaning transform in dialogue.

### 6.2 Limitations

Precisely because it imposes structure, the *Dimensional Model* inevitably excludes what resists formalization. Any segmentation of therapy into discrete units simplifies the fluid, co-created nature of human dialogue. The model therefore clarifies mechanisms of movement but cannot capture the emergent, non-rule-bound transformations that constitute the art of therapy.

Another limitation concerns scope: the framework currently addresses the organization of psychic discourse, not its developmental, somatic, or neurobiological substrates. Nor does it account for the cultural or linguistic variability that may shape dimensional expression. These boundaries are not flaws but define the epistemic domain of the model: a map of mental functioning, not a map of mind in total. Future elaborations may extend the architecture to include embodied, temporal, and social dimensions, but within its present formulation, the model should be judged by its explanatory precision, not its comprehensiveness.

### 6.3 Conceptual boundaries and future directions

Beyond these practical considerations, the model carries inherent conceptual constraints embedded within each of its structural components (the five dimensions, the cascade, and their operational principles). Future development may involve expanding the model to encompass additional domains such as body, time, intersubjectivity, or even chaos. These could help delineate the model’s boundaries and reveal what stirs between or destabilizes its dimensions, thereby enriching our understanding of psychic motion beyond its current architecture.

Similarly, the *Dimensional Cascade* may include yet unrecognized transitional zones, fractures, or peripheral dimensions that influence inner equilibrium and the system’s capacity for self-renewal. Likewise, further inquiry may uncover latent or implicit principles of operation that remain outside the current framework but subtly shape the flow between dimensions, challenge continuity, or stimulate dynamic reorganization.

Taken together, these limitations should not be regarded as weaknesses but as generative invitations. They call for continued conceptual refinement, empirical exploration, and methodological innovation in testing and expanding the *Dimensional Model* of the psyche.

Recognizing these limits clarifies the model’s status: not a closed system of explanation, but an analytic lens whose strength lies in the disciplined articulation of what can be observed, rather than in the promise of theoretical totality. Its primary contribution may therefore lie in offering a shared conceptual grammar through which diverse psychological processes can be rendered intelligible within a single structural vocabulary.

### 6.4 Theoretical context and distinction

Efforts to integrate psychology’s disparate paradigms are not new. Henriques’s Tree of Knowledge framework Henrique’s, 2004, 2011 proposed a hierarchical unification of natural and social sciences through levels of complexity, situating psychology as the “joint point” between biology and culture. The *Dimensional Model* shares this integrative ambition but differs in scope and focus: rather than describing a phylogenetic hierarchy of knowledge systems, it delineates the functional architecture of the psyche itself, the dynamic transitions through which subjective life unfolds from *being* to *action*. Its concern is phenomenological and processual rather than epistemological or disciplinary.

Hayes and Hofmann’s (2021) Process-Based Therapy (PBT) movement represents a second major integrative current, replacing schools of therapy with empirically defined change processes. The *Dimensional Model* complements this approach by supplying a meta-structural grammar of those processes. Where PBT enumerates mechanisms of change (e.g., cognitive flexibility, attentional control), the Dimensional framework articulates the underlying configurations of mental functioning within which such mechanisms operate and transform. It thus offers a phenomenological topology to accompany the nomothetic taxonomy of PBT.

Dynamic-systems formulations (e.g., Schiepek and Tschacher, 2020) likewise emphasize self-organization, feedback, and attractor states in psychotherapy. The *Dimensional Model* converges with this perspective in portraying the psyche as a non-linear system governed by principles of *equilibrium* and *flexibility*, yet diverges by grounding these dynamics in experiential and semantic rather than purely mathematical terms. It translates systemic regulation into clinically observable movements between dimensions.

Finally, Damasio’s (2021) neurophenomenological synthesis underscores the hierarchical emergence of mind from bodily regulation to consciousness. The *Dimensional Model* parallels this ascending organization but extends it beyond neural description to the symbolic and intersubjective field, framing the same continuity from a first-person psychological standpoint.

In this respect, the *Dimensional Model* positions itself as a bridging meta-framework: not a replacement for these integrative paradigms but a phenomenologically precise matrix linking subjective experience, clinical practice, and empirical observation within a single, dynamically regulated structure of mind.

## Data Availability

The original contributions presented in this study are included in this article/supplementary material, further inquiries can be directed to the corresponding author.
